# Beneficial Effect of Phenytoin and Carbamazepine on *GFAP* Gene Expression and Mutant GFAP Folding in a Cellular Model of Alexander’s Disease

**DOI:** 10.3389/fphar.2021.723218

**Published:** 2021-12-07

**Authors:** Tiziana Bachetti, Eleonora Di Zanni, Annalisa Adamo, Francesca Rosamilia, M. Margherita Sechi, Paolo Solla, Matteo Bozzo, Isabella Ceccherini, GianPietro Sechi

**Affiliations:** ^1^ UOSD Laboratorio di Genetica e Genomica delle Malattie Rare, IRCCS Gaslini, Genova, Italy; ^2^ Laboratorio di Neurobiologia dello Sviluppo, DISTAV, Università di Genova, Genova, Italy; ^3^ Dipartimento di Scienze della Salute, DISSAL, Università di Genova, Genova, Italy; ^4^ Department of Medical Sciences and Public Health, University of Cagliari, Cagliari, Italy; ^5^ Department of Medical, Surgical and Experimental Sciences (G.P.S.; P.S.), University of Sassari, Sassari, Italy

**Keywords:** glial fibrillary acid protein, clomipramine, carbamazepine, phenytoin, Alexander disease

## Abstract

Alexander’s disease (AxD) is a rare, usually relentlessly progressive disorder of astroglial cells in the central nervous system related to mutations in the gene encoding the type III intermediate filament protein, glial fibrillary acidic protein (GFAP). The pathophysiology of AxD is only partially understood. Available data indicate that an excessive GFAP gene expression may play a role. In particular, a “threshold hypothesis” has been reported, suggesting that mutant GFAP representing about 20% of the total cellular GFAP should be sufficient to cause disease. Thus, strategies based on reducing cellular mutant GFAP protein levels and/or activating biological processes involved in the correct protein folding could be effective in counteracting the toxic effect of misfolded GFAP. Considering that clomipramine (CLM), which has been selected by a wide small molecules screening as the greatest inhibitory potential drug against GFAP expression, is contraindicated because of its proconvulsant activity in the infantile form of AxD, which is also characterized by the occurrence of epileptic seizures, two powerful antiepileptic agents, carbamazepine (CBZ) and phenytoin (PHT), which share specific stereochemical features in common with CLM, were taken into consideration in a reliable *in vitro* model of AxD. In the present work, we document for the first time that CBZ and PHT have a definite inhibitory effect on pathological GFAP cellular expression and folding. Moreover, we confirm previous results of a similar beneficial effect of CLM. In addition, we have demonstrated that CBZ and CLM play a refolding effect on mutant GFAP proteins, likely ascribed at the induction of CRYAB expression, resulting in the decrease of mutant GFAP aggregates formation. As CBZ and PHT are currently approved for use in humans, their documented effects on pathological GFAP cellular expression and folding may indicate a potential therapeutic role as disease-modifying agents of these drugs in the clinical management of AxD, particularly in AxD patients with focal epilepsy with and without secondary generalization.

## Introduction

Alexander’s disease (AxD, MIM#203450) is a rare, usually relentlessly progressive genetic disorder of astroglial cells in the central nervous system related to heterozygous mutations in the gene encoding the type III intermediate filament protein, glial fibrillary acidic protein (GFAP) (Brenner et al., 2001). Two main clinical subtypes are generally recognized (Balbi et al., 2010; Prust et al., 2011): AxD type I has an infantile presentation with encephalopathy, cognitive impairment, epilepsy, and failure to thrive; AxD type II has a juvenile/adult onset and commonly presents with bulbar dysfunction (e.g., dysarthria, dysphonia, and palatal myoclonus), ataxia, and spastic paraparesis. Compared to the infantile form, AxD type II has a slower progression and cognitive impairment is usually absent (Balbi et al., 2010; Prust et al., 2011). The pathological hallmark of AxD is the occurrence of intracytoplasmic eosinophilic aggregates in astrocytes (Rosenthal fibers), containing GFAP, the small heat shock proteins (sHSPs), alphaB-Crystallin and HSP27, and ubiquitin (Mignot et al., 2004; Hsiao et al., 2005). The pathophysiology of AxD is only partially understood. Available data indicate that an excessive GFAP gene expression may play a role as indicated by experimental mouse models overexpressing GFAP (Hagemann et al., 2005). In particular, a “threshold hypothesis” has been reported, suggesting that mutant GFAP representing about 20% of the total cellular GFAP is sufficient to cause disease (Hagemann et al., 2005). Thus, molecular mechanisms regulating brain GFAP levels could represent molecular targets, too (Cho et al., 2010).

In addition, autophagy-mediated mutant protein elimination and sHSPs-mediated refolding are considered druggable molecular targets, whose modulation, in *in vivo* and *in vitro* experimental model of AxD, has been documented to result in the rescue of filamentous mutant GFAP patterns (Bachetti et al., 2010a; Bachetti et al., 2012). In particular, alphaB-crystallin may act as a chaperone for the correct folding of cytoskeletal proteins and it is upregulated following different types of cellular stress (Hagemann et al., 2009). alphaB-Crystallin has been shown to be active in decreasing GFAP mediated toxicity. Specifically, in AxD mouse models overexpressing wild-type GFAP (e.g., mice transgenic for R236H mutation) and in double tg (GFAP^Tg^;GFAP^+/R236H^), alphaB-crystallin overexpression was able to decrease the stress response in the central nervous system and increase animal survival (Hagemann et al., 2009).

Although to date there are no definite treatments or disease-modifying agents available for this neurodegenerative disorder, recent clinical data (Sechi et al., 2010; Sechi et al., 2013) and findings from *in vitro* and animal models of AxD indicate that drugs commonly used in clinical practice such as the beta-lactam antibiotic ceftriaxone and the tricyclic antidepressant clomipramine (CLM) may have a therapeutic role (Bachetti et al., 2010b; Cho et al., 2010).

Clomipramine, in particular, has been selected by a wide small molecules screening to identify drugs with the greatest inhibitory potential against GFAP expression (Cho et al., 2010). Notably, this drug has also been observed to be effective in reducing GFAP expression in the hippocampus after chronic unpredictable stress in a rat model of depression (Liu et al., 2014). Clomipramine has been used for decades to treat depression, obsessive-compulsive disorder, and panic attacks (Trimble, 1990; Sayyah and Rahim, 2018). However, a major disadvantage for the long-term use of this drug is the increased risk of reactive epileptic seizures, mainly in patients with a history of neurodegeneration and a low seizure threshold (Alper et al., 2007). Since the infantile form of AxD is frequently characterized by the occurrence of epileptic seizures (Li et al., 2005), ideally, it should be advisable that compounds able to suppress or modulate GFAP expression may also have antiepileptic activity.

**FIGURE 1:**
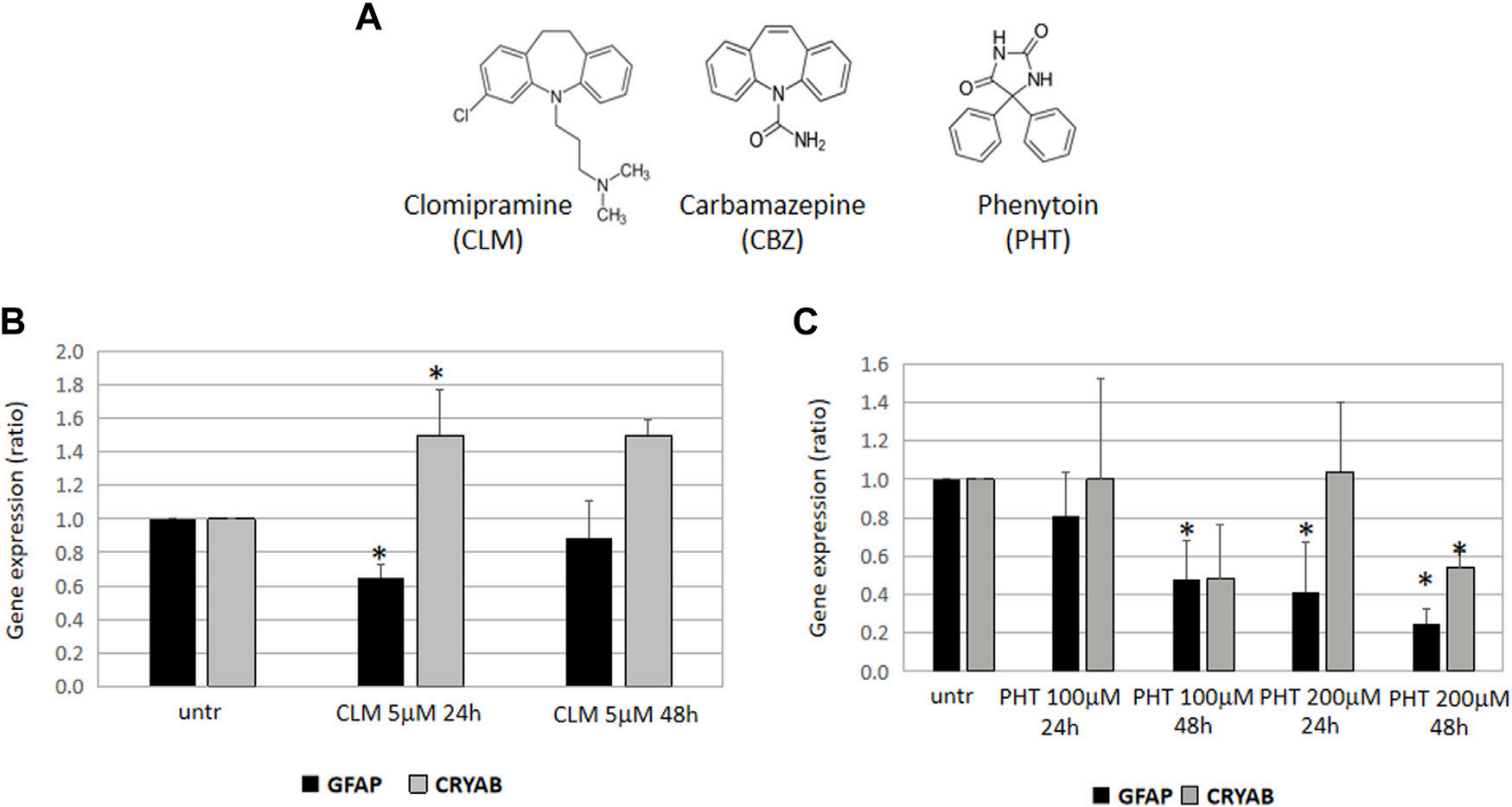
Effects of CLM, PHT, and CBZ on GFAP expression and folding. **(A)** Chemical structures of clomipramine, carbamazepine, and 5,5-diphenylhydantoin. Carbamazepine (CBZ) is structurally related to the tricyclic antidepressant clomipramine (CLM) and has specific stereochemical features in common with 5,5-diphenylhydantoin (PHT). GFAP and CRYAB expression in U251-MG cells treated with CLM 5 mM **(B)** or PHT 100 and 200 nM **(C)** for 24 and 48 h. All the diagrams show the expression of GFAP and alphaB-crystallin (CRYAB), represented as fold induction with respect to untreated samples (arbitrary value = 1). Values are the mean ± SD of three independent experiments performed in triplicate.

Considering that carbamazepine (CBZ), a commonly used antiepileptic agent, is an iminostilbene derivative with a dibenzazepine nucleus and thus has a tricyclic structure similar to clomipramine (Figure 1A) (Sillanpää et al., 1998), and that phenytoin (PHT) (named also 5,5 diphenylhydantoin), another powerful antiepileptic agent, although having a little chemical resemblance, has specific stereochemical features in common with carbamazepine (Figure 1A) (Jones et al., 1981), we thought it would have been extremely relevant to investigate the effects of these antiepileptic compounds on *GFAP* mRNA expression and GFAP folding in an *in vitro* model of AxD.

## Materials and Methods

### Cell Cultures and Treatments

The U251-MG human astrocytoma cell line was grown in RPMI medium supplemented with 10% FBS (Gibco, New Zealand), 1% Penicillin, and Streptomycin (Euroclone). Carbamazepine (CBZ, Sigma) was prepared as a 2.1 mM stock solution in EtOH; clomipramine hydrochloride (CLM, Sigma) was prepared as a 100 mM stock solution in water; 5,5-diphenylhydantoin (PHT, Sigma) was prepared as a 100 mM stock solution in DMSO. Drugs were added at concentrations currently used and already reported to display effects on pathways whose modulation may affect GFAP expression. In particular, final concentrations of 5 and 30 μM of CLM and 100 and 200 μM of CBZ and PHT were applied to cell cultures according to their effects in similar ranges of concentrations in other models on gene transcription or autophagy induction (Table 1). Analyses were performed after 24 and 48 h treatment. Bafilomycin A1, 160 μM stock, was freshly diluted in culture medium to the final 100 nM working solution.

**TABLE 1:** FACS analysis of GFAP WT and p.R239C-GFP level.

Sample	MFI (%untreated = 100)	Sample	MFI (% untreated = 100)
WT unt	30.5 (100%)	WT unt	36.7 (100%)
WT + CBZ 30 μM	29.9 (98%)	WT + CLM 5 μM	38.73 (105%)
R239C unt	30.01 (100%)	R239C unt	36.19 (100%)
R239C + CBZ 30 μM	29.6 (98%)	R239C + CLM 5 μM	34.98 (96%)

### Expression Constructs and Transient Transfections

Expression constructs encoding GFAP WT and GFAP carrying the p. R239C mutation fused, at the C-terminal, with the green fluorescent protein (GFP) have already been described (Bachetti et al., 2010a). Transient transfections were performed in U251-MG cells plated in 35 mm Amniodishes with 1 μg of the expression vectors or alternatively in four-well chamber slides with 300 ng plasmids, using Lipofectamine 2000 (Invitrogen) 3:1 following manufacturer’s instructions. Drugs were added to cell cultures the day after transfection and samples were assayed for gene expression analysis or fluorescence assays after 24 or 48 h.

For LC3B dots detection, 300 ng of the pEX- LC3B HcRed plasmid was transfected into four-well chamber slides in the absence or presence of bafilomycin 100 nM for the last 4 h before microscope fluorescence analysis.

### Gene mRNA Expression Analysis

Total RNA was extracted from U251-MG cells untreated and treated with drugs (“RNeasy Mini Kit,” Qiagen) and cDNA synthesized (iScript cDNA Synthesis Kit”, BioRad) following the manufacturer’s instructions. Gene expression analysis of *GFAP* and *CRYAB* genes has been performed as already reported (Bachetti et al., 2012). Normalization was obtained using G3PDH and beta2-microglobulin housekeeping genes. Values are the mean of at least three independent experimental triplicates.

### GFAP Promoter Activity

U251-MG cells were transfected with a construct containing the *GFAP* promoter consisting of 2,2 Kb cloned upstream of the *luciferase* gene (Bachetti et al., 2010b) and treated with drugs for 24 h or 48 h. Luciferase activity was measured by a TD 20/20 luminometer following the manufacturer’s instructions (Bachetti et al., 2010a). Values are the mean of at least three independent experimental triplicates.

### Western Blot Analysis of Endogenous GFAP Protein

U251-MG cells were plated in 60 mm dishes and were treated with drugs. After 24 h, cells were washed with PBS 1× and centrifuged and lysed with RIPA buffer (Tris–HCl 50 mM pH 7.5, NaCl 150 mM, Triton-X 1%, SDS-20 0.1%, Na deoxycholate 1%, protease Inhibitor mix 1× ). Total cell lysates were quantified and equal amounts were electrophoresed on 10% SDS-PAGE and transferred to a polyvinylidene difluoride membrane.

Proteins were identified by probing the membrane with the mouse anti-GFAP (Cell Signaling), anti-alphaB-Crystallin (Cell Signaling), and anti-tubulin antibodies (Sigma-Aldrich). Signals were detected using the chemiluminescence reagent ECL advance (Amersham) and protein levels in each sample were evaluated by comparison with corresponding amounts of the housekeeping tubulin. Densitometric analysis of proteins was performed by ImageJ software. For each sample, GFAP and anti-alphaB-crystallin amount was evaluated following normalization on the housekeeping tubulin.

### Fluorescence Microscopy Analysis

For immunofluorescence, U251-MG cells were plated in four-well chamber slides and treated for 24 h with drugs. Cells were then fixed with MeOH:acetone 1:1, permeabilized for 10′ with PBS/0.1% Triton-x-100, and blocked for 5 min with PBS/FBS 5%. Cells were stained with GFAP antibody (Cell Signaling, mouse (GA5) #3670S, 1:250) for 2 h or with anti-vimentin (Sigma-Aldrich, #HPA0001762, lot. B114381, 1:250) followed by 1 h incubation with AlexaFluor 555 anti-mouse or AlexaFluor 568 anti-rabbit secondary antibody (1:800). Images were acquired by Zeiss fluorescence microscope equipped with a camera and acquired by Nikon ACTU software. To allow comparison between samples, 1.5″ acquisition time was used for each condition.

For evaluation of exogenous GFAP conformation, U251-MG cells were plated in Amniodishes (150.000 cells/sample) and transfected with 1 μg expression plasmid coding for p. R239C-GFAP-GFP. After 24 h , cells were treated with drugs and 48 h after transfection analyzed by fluorescence microscope.

In both conditions, nuclei were counterstained with DAPI. Drug effect was evaluated based on their ability to decrease the number of intracytoplasmic GFAP-GFP aggregates, detected as dense optical spots that could be clearly recognized over or instead of the GFAP filaments.

In particular, cells were classified into three patterns: a pattern exclusively formed by filaments (F), a pattern exclusively formed by aggregates (A), and a mixed pattern with filaments and aggregates (F + A).

### Flow Cytometry Analysis of GFAP-GFP Protein Levels

Transient transfections were performed plating, directly in 60 mm diameter dishes, 3 × 10^5^ U251-MG cells with the transfection mix containing 2 μg of either GFAP WT-GFP or p. R239C-GFP expression constructs and 6 μL of Lipofectamine 2000 (Invitrogen). Twenty-four hours after transfection, cells were treated with drugs; 24 h later, cells supernatant was removed and cells were trypsinized and collected in a 5 ml culture medium. Following an already reported procedure (Bachetti et al., 2008), cells suspensions were analyzed by fluorescence cytometry (FACSCalibur, BD Biosciences, Cell Quest software) by acquiring the same number of events represented by positive GFP cells expressing transfected GFAP proteins.

On these populations, the mean fluorescence intensity (MFI), representing the amount of GFP, was assessed as a measure of exogenous GFAP. For both GFAP WT and R239C, a comparison between MFI from untreated and treated samples was performed and values were expressed as a percentage of untreated cells.

### Statistical Analysis

For each assay, at least three independent experiments were performed. Statistic significant differences between untreated and treated samples were evaluated by applying paired Student’s *t*-test (*p* value < 0.5). For fluorescence microscope analysis, this comparison was performed between cells with the same pattern (F, F + A, A).

## Results

### Effects of Drugs on Endogenous Expression of GFAP and alphaB-Crystallin Genes

As GFAP overexpression is considered a main pathogenetic determinant in AxD leading to GFAP accumulation, and the small heat shock proteins (sHSPs) overexpression a potential endogenous tool to counteract GFAP misfolding and cellular protein accumulation, the expression of GFAP and alphaB-Crystallin has been investigated in U251-MG cells after 24 and 48 h treatments.

We have observed that 24 h treatment with CLM was able to downregulate GFAP and to upregulate alphaB-crystallin expression, thus suggesting that this drug may have a beneficial effect by activating at least two definite metabolic pathways involved in mutant GFAP accumulation (Figure 1B).

Moreover, a period of 24 hours was also sufficient for the highest PHT dose to downregulate *GFAP* expression, an effect even more evident after a 48 h treatment. However, at the same time and dose conditions, alphaB-crystallin was downregulated, thus suggesting that, in the presence of mutant GFAP proteins, PHT might have a beneficial effect only by decreasing cytoplasmic GFAP but not by enhancing a correct folding mediated by alphaB-crystallin (Figure 1C). Unfortunately, the effect of CBZ could not be tested; although the quality of the RNA and cDNA samples was good, the CBZ solvent ethanol caused technical issues during the real-time PCR assay and undetectable values in all conditions did not allow any downstream evaluation.

### Effects of Drugs on the GFAP Promoter Activity

To investigate whether the effects played by CLM and PHT on GFAP mRNA were mediated by transcriptional regulation, we have transfected cells with a construct containing the GFAP promoter cloned upstream the luciferase gene and performed a transactivation analysis, as previously reported (Bachetti et al., 2010b; Bachetti et al., 2012).

**FIGURE 2:**
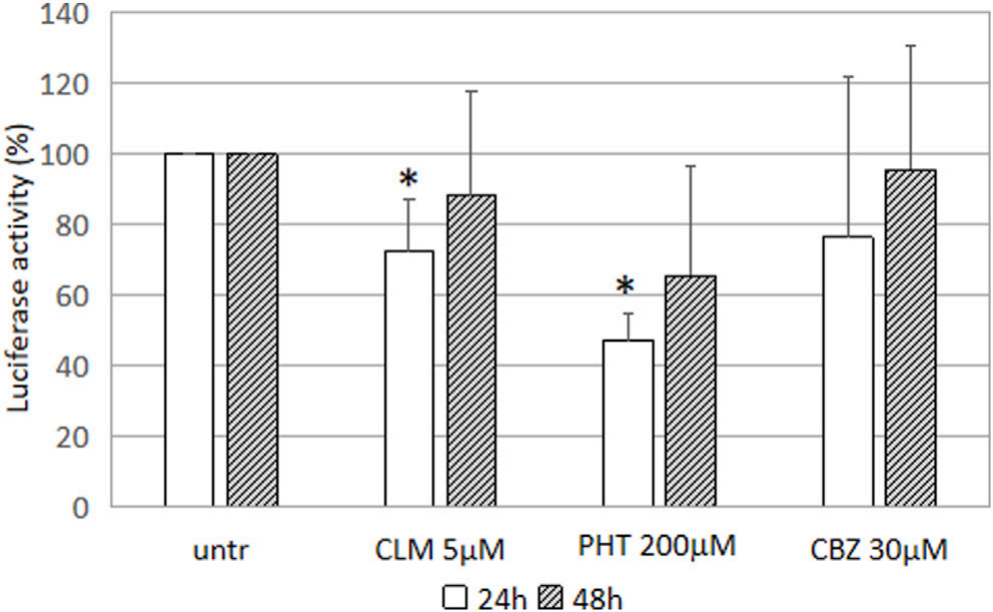
Effect of the drugs on GFAP transcription luciferase activity driven by GFAP promoter in U251-MG cells treated with CLM 5 mM. Values of luciferase activity have been expressed as the percentage of untreated cells and are the mean ± SD of three independent experiments performed in triplicate. Asterisks indicate statistically significant values (*p < 0.05).

The highest PHT 200 μΜ dose was able to reduce the *GFAP* promoter activity after 24 h and 48 h s, in accordance with experimental conditions already known to be effective on *GFAP* mRNA. Similarly, also CLM showed a significant effect on promoter downregulation, in accordance with results obtained *in vivo* experimental models (Cho et al., 2010). CBZ, instead, was not able to modulate GFAP transcription in our cellular model and under test conditions (Figure 2).

### Effects of Drugs on the GFAP and AlphaB-Crystallin Proteins

**FIGURE 3:**
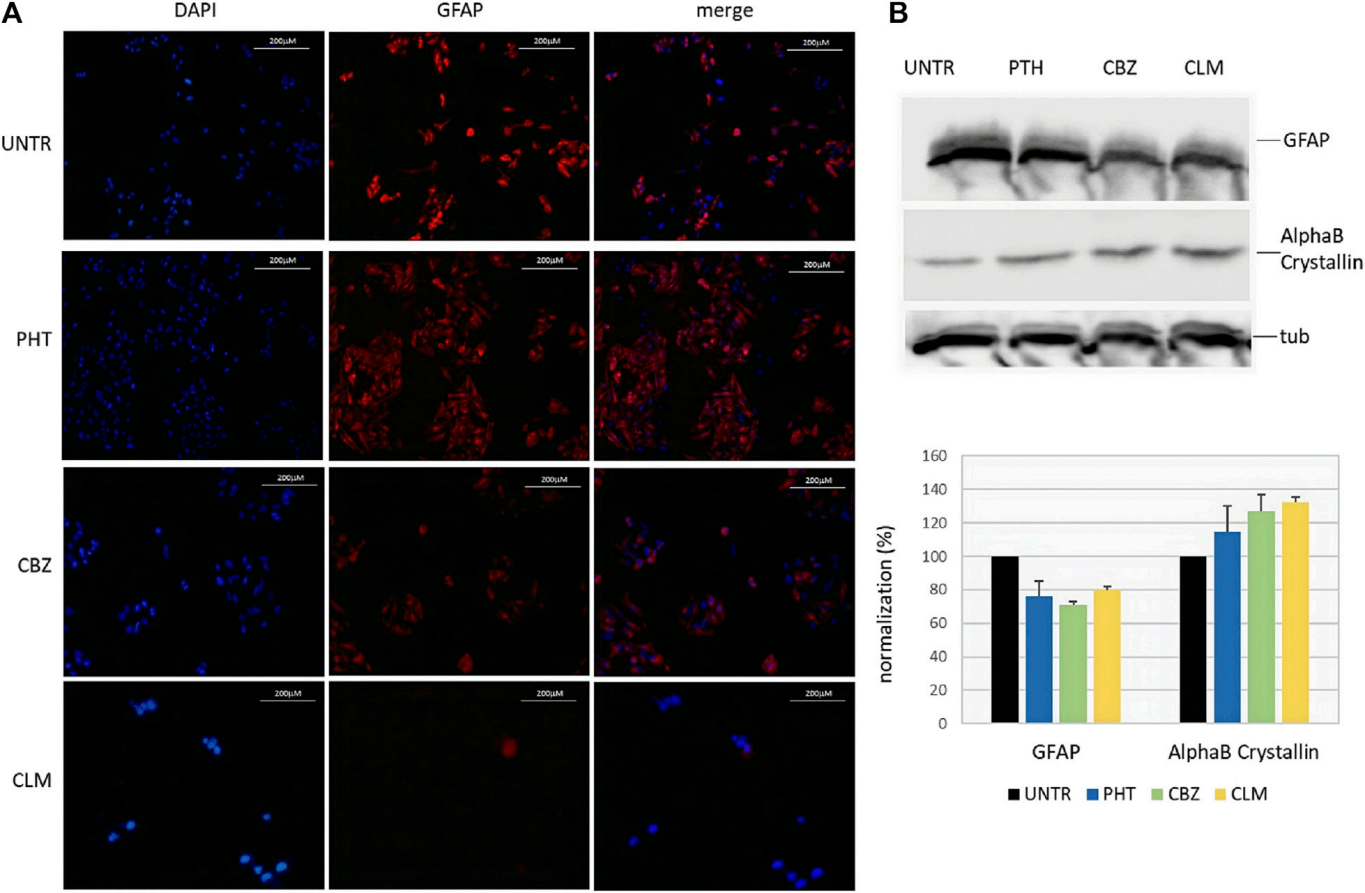
Effect of the drugs on endogenous GFAP and alphaB-crystallin proteins. **(A)** Immunofluorescence of endogenous GFAP following treatments in U251-MG cells. From left to right, nuclei (DAPI), anti-GFAP, and composite images (merge) are shown in untreated (upper line) and following 24 h treatments with PHT, CBZ, and CLM. Cells were analyzed by a Zeiss fluorescence microscope equipped with ×20 magnification objective; images of each condition were acquired at the same exposure time. **(B)** Western blot of endogenous GFAP and alphaB-crystallin proteins following treatments. Expression of the target proteins and the housekeeping tubulin was analyzed in lysates from U251-MG cells untreated and added with PHT and CBZ. In the bottom image, quantification of GFAP and alphaB-crystallin proteins following normalization on tubulin is shown for each sample in a bar diagram. Values are the mean of two independent experiments±SD.

To assess whether drug-mediated effects on mRNA could be confirmed at the protein level, we have evaluated endogenous GFAP and alphaB-crystallin levels in U251-MG cells following 24 h drug treatments. In particular, in addition to PHT and CLM, already observed to play a role in regulating the mRNA levels, we have also tested CBZ because we could not exclude its role in the posttranscriptional GFAP regulation. First, in immunofluorescence assay performed with specific GFAP antibody, we have observed that, with respect to untreated cells showing almost all an intense red signal, a significant proportion of PHT treated cells was characterized by less intense red fluorescence. Following CBZ and CLM treatments, signals further decreased, thus suggesting that these two drugs are the most effective in decreasing GFAP protein levels (Figure 3A). Unfortunately, immunofluorescence analysis could not produce a detectable signal for alphaB-crystallin suitable to perform quantitative evaluations.

Therefore, western blot was carried out to quantitatively confirm the qualitative results obtained by immunofluorescence. Lysates from cells treated with PHT, CBZ, and CLM confirmed the results obtained by immunofluorescence. Normalization of GFAP and alphaB-crystallin protein levels using the housekeeping tubulin confirmed observations derived from immunofluorescence (Figure 3B, upper part).

In particular, these results show that CLM and PHT treatments resulted in downregulation not only of the GFAP mRNA but also of the GFAP protein (Figure 3B, bottom part). In addition, they suggest that also CBZ is able to reduce GFAP protein levels, although we could not evaluate its effects on mRNA.

Moreover, CBZ and CLM showed a reproducible effect in increasing alphaB-crystallin protein, while the effect of PHT was more variable and, for this reason, considered not significant.

### Effects of Drugs on Mutant GFAP Aggregates

**FIGURE 4:**
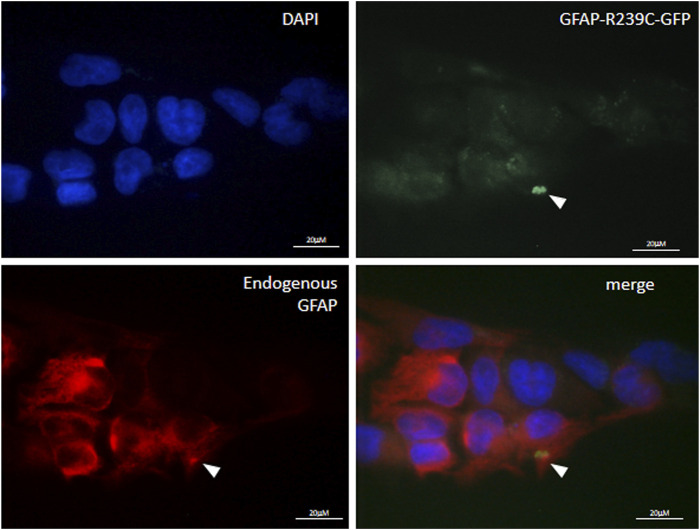
Characterization of p. R239C GFAP immunofluorescence of endogenous GFAP in U251-MG cells expressing exogenous p. R239C-GFP. From top left to bottom right, nuclei (DAPI), green exogenous p. R239C-GFP, red endogenous GFAP, and merged images are shown. Cells were analyzed by a Zeiss fluorescence microscope equipped with1×00 magnification objective.

**FIGURE 5:**
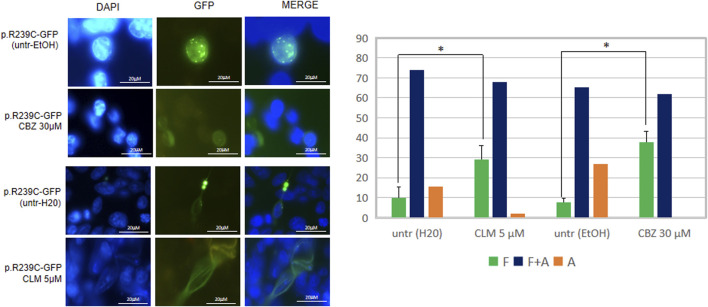
Analysis of drugs’ effect on GFAP cytoskeletal organization. Fluorescence microscope images represent examples of the conformation of the transfected mutant GFAP protein in U251-MG cells without any treatment (untr) or in the presence of the doses indicated of CBZ and CLM. From left to right, DAPI-stained nuclei, GFP-fused GFAP fluorescence detection, and a merge of the two stainings are reported, respectively. The diagram below showed the percentage of cells characterized by filaments (F), filaments and aggregates (F + A), and only aggregates (A) in the absence and presence of the above molecules and expressed as the proportion of transfected and untreated cells showing only GFAP filaments. Values are the mean ± SD of three independent experiments. Statistical analysis for F patterns has been performed by Student’s *t*-test [CLM vs. untr H2O (F): *p* = 0.003; CBZ vs. untr EtOH (F): *p* = 0.019].

**FIGURE 6:**
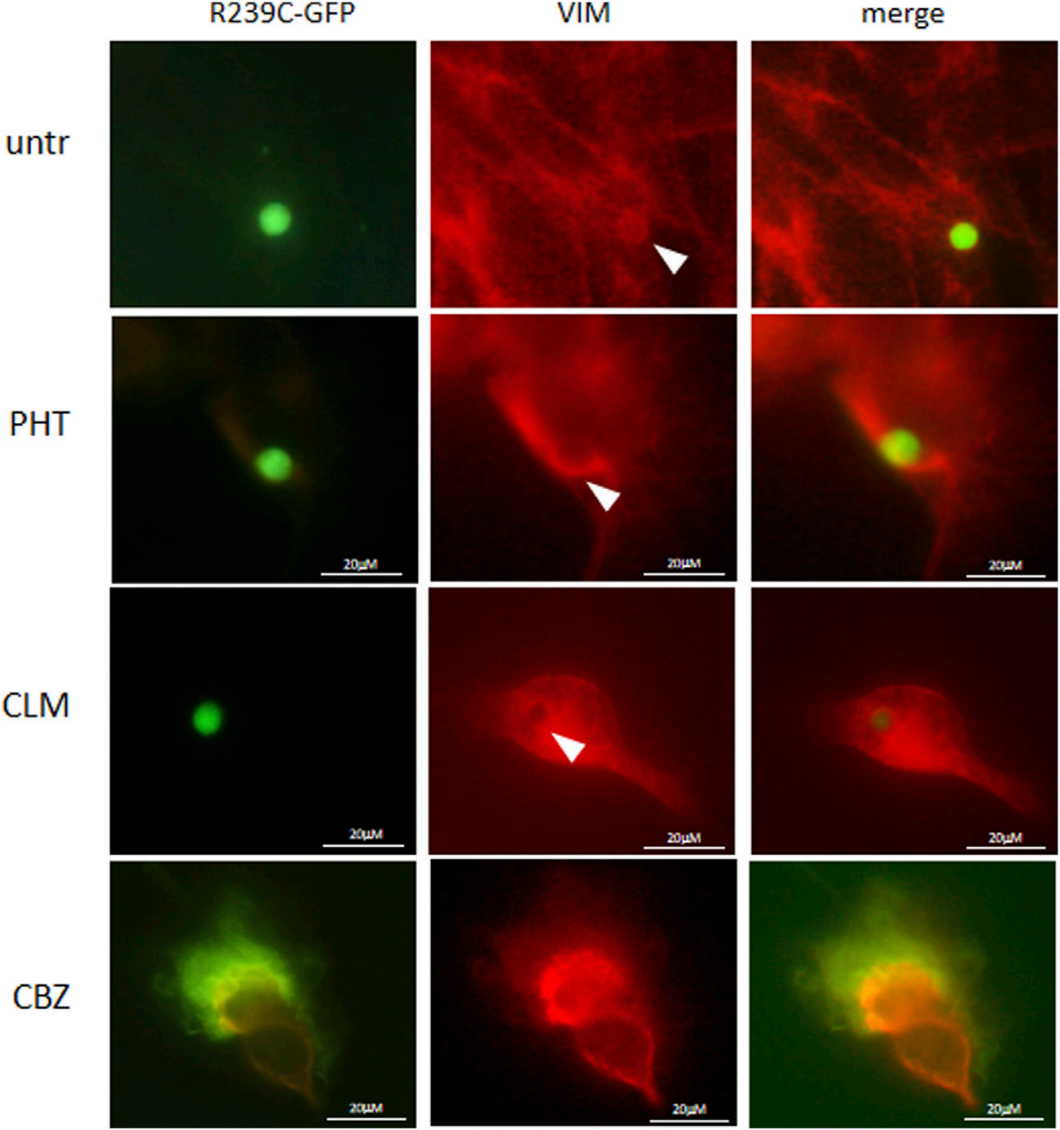
Analysis of drugs’ effect on vimentin cytoskeletal organization. Fluorescence microscope images show vimentin distribution in untreated (untr) and PHT-, CLM-, and CBZ-treated cells. Cells were analyzed by a Zeiss fluorescence microscope equipped with100X magnification objective.

We have also investigated whether the pathological folding of the mutant p. R239C-GFAP protein, transiently expressed in U251-MG cells, could be at least partially counteracted by the addition of the molecules selected on the basis of results observed in our previous experiments. First, in order to confirm the suitability of this cellular model, we assessed that GFP dots do contain GFAP (Figure 4) and PHT was not tested because of its uncertain effect on alphaB-crystallin expression (Figures 1C, 3B). Conversely, CLM and CBZ were tested because they act as an inducer of alphaB-crystallin gene expression (Figures 1B, 3B). As shown in Figure 5, we found that both CLM and CBZ appeared to be effective in inducing a decrease in cellular GFAP aggregates in parallel with a corresponding expected increase in normal GFAP filament formation. Moreover, cells still showing some aggregates after treatments were analyzed for vimentin distribution. Immunofluorescence analysis confirmed that in the presence of GFAP perinuclear inclusion, vimentin was found in the aggresomes, as shown after PHT and CLM treatments. On the other hand, a regular vimentin distribution was observed in the presence of a GFAP filamentous pattern, as shown after CBZ treatment (Figure 6).

**FIGURE 7:**
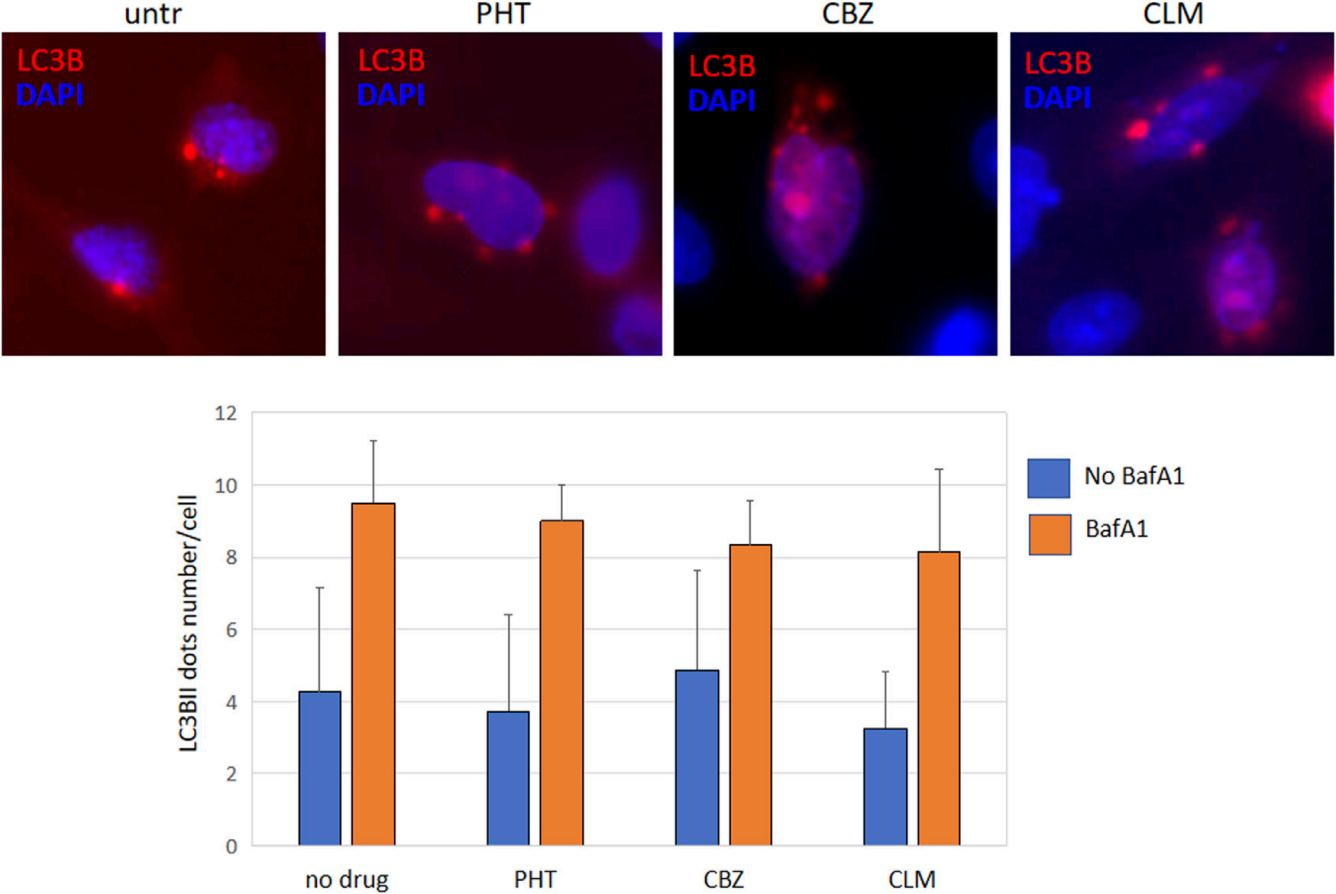
Autophagy evaluation images show U251-MG cells transfected with pEX-LC3B-HcRed and treated with PHT, CBZ, and CLM. Red dots represent lipidated LC3BII lying in the autophagosome membranes; nuclei were counterstained with DAPI (blue). Each sample was also evaluated in the presence of bafilomycin A1 100 nM for 4 h to monitor if there were differences in the autophagic flux. The bar diagram below represents the mean ± SD of dots number in each condition, evaluated in 50 transfected cells.

Finally, quantitative analysis of intracellular R239C-GFAP-GFP aggregates, performed to investigate if the refolding mediated by drugs could be partly due to the natural mutant protein elimination, allowed us to exclude a significant spontaneous decrease in cellular protein levels in the timeframe of drug treatments (Table 1). These results were further confirmed by transfection of a plasmid encoding the autophagy marker LC3B fused to HcRed, following a protocol already set up in our lab (Bianchi et al., 2018); evaluation of dots formation, corresponding to the lapidated LC3BII form lying on the autophagosome membrane and indicating active autophagy, allowed to exclude any differences in autophagy extent between untreated and treated cells (Figure 7). Overall, this finding suggests that, in our experimental model, normal filaments formation should be ascribed primarily to HSPs mediated refolding rather than to spontaneous decreased levels of mutant GFAP. However, we cannot exclude further still undisclosed mechanisms that could take part in drug effects.

## Discussion

In the present work, we have documented for the first time that PHT and CBZ, two commonly used antiepileptic drugs worldwide, have an inhibitory effect on pathological GFAP cellular expression and folding in a reliable *in vitro* model of AxD. These findings may suggest a potential therapeutic role of these antiepileptic agents in the management of AxD in clinical practice, including the infantile form of AxD, which is also characterized by a low seizure threshold and the frequent occurrence of epileptic seizures (Li et al., 2005; Balbi et al., 2010). Moreover, we confirm previous results of a definite beneficial effect of CLM in *in vitro* and animal models of AxD (Cho et al., 2010). On the other hand, differently from CBZ and PHT, CLM also has a potent proconvulsant activity that limits its chronic use in patients with AxD, especially in the infantile form of the disease.

The effects of CLM, PHT, and CBZ have successively been tested on cellular pathways involved in refolding of mutant GFAP proteins on *GFAP* gene expression and mutant GFAP conformation.

In particular, to investigate the mechanisms of action of these compounds on cellular biochemical pathways involved in GFAP synthesis and folding, we have first assayed the effect of CLM and PHT on endogenous expression of GFAP and the expression of the sHSP alphaB-crystallin whose level is crucial for the correct folding and assembly of cytoskeletal filaments in astrocytes. CLM decreased the expression of endogenous *GFAP* mRNA, increasing at the same time the expression of the sHSP alphaB-crystallin, while PHT displayed only a partial beneficial effect by decreasing GFAP mRNA but not by enhancing its correct folding through alphaB-crystallin. In our cellular model, only PHT has demonstrated that the reduced mRNA level was due to a significant downregulation of the *GFAP* promoter, which is consistent with the decrease of *GFAP* transcript already detected by real-time PCR. In addition, all drugs ultimately exerted a downregulation effect on GFAP protein levels, although at a different extent. Moreover, both CLM and CBZ have been shown to induce the formation of filaments in cells expressing the p. R239C mutant GFAP, a beneficial effect likely resulting from induction of mutant protein refolding, as suggested by CLM mediated sHSP upregulation. In particular, CBZ has been revealed to be the most efficient treatment in rescuing the correct pattern of mutant GFAP, thus suggesting that its effect may be mediated mainly by HSPs response rather than by eliminating the mutant protein. Nevertheless, the involvement of cellular mechanisms facilitating the elimination of mutant GFAP proteins has yet to be definitively demonstrated. Furthermore, as cells transfected and treated with CBZ and CLM do not show a significant GFAP reduction compared to untreated cells, it can be hypothesized that their beneficial effect on filaments formation may be exclusively mediated by increased refolding, likely due to HSPs induction rather than to ubiquitin-proteasome system and/or autophagy activation. In particular, regulation of autophagy was excluded by monitoring the autophagic flux through the evaluation of LC3B dots in the presence of inhibition of autophagosome-lysosome fusion (Klionsky et al., 2016) as quantification of LC3B dots did not show any differences between drug treatments.

However, we cannot exclude that also PHT, although not inducing sHSPs, in physiological models could facilitate mutant GFAP folding by reducing its mRNA levels. Such a hypothesis is based on our previous observations that there is a correlation between decreased levels of transfected mutant GFAP and filaments assembling (Bachetti et al., 2010a). However, this possibility could not be assessed in our cellular model as p. R239C expression was driven by a viral promoter of the plasmid upstream of the GFAP coding region. Moreover, based on the same principle, we have identified a novel mechanistic role of CLM since its ability in inducing a correct folding, in the present model, seems independent of downregulation of *GFAP* expression.

In agreement with the targetable pharmacological mechanisms known to be effective in AxD, the small molecules taken into consideration in the present work seem to act mainly by modulation of GFAP gene expression and induction of HSPs such as αB-crystallin, as summarized in Table 2. However, other yet unidentified molecular mechanisms might also be involved and deserve to be mentioned, such as a direct chaperone-like effect of PHT, CBZ, and CLM on correct three-dimensional GFAP folding. Of note, our group recently documented such a possibility for PHT and ceftriaxone by spectroscopic experimental studies (Ruzza et al., 2016).

**TABLE 2:** Reported effects of drugs at given concentrations.

Drug	Concentration	Effect	Reference
Clomipramine (CLM)	5–10 μM	Reduction of GFAP expression	Cho et al., 2010; Liu et al., 2014
Carbamazepine (CBZ)	30 μM 120 mg/ kg	1. Autophagy induction 2. reduced GFAP immunolabelling	Hidvegi et al., 2010; Cunha et al., 2009
5,5-Diphenylhydantoin (PHT)	100–200 μM 60 mg/ kg	Transcriptional regulation reduced GFAP immunolabelling	Koide et al., 2009; Cunha et al., 2009

Overall, in terms of beneficial effects useful for a possible pharmacological treatment of AxD, results obtained in our cellular system can be summarized as follows: PHT can decrease GFAP expression; CBZ and CLM can decrease GFAP expression and increase folding, mediated by upregualtion of alphaB-crystallin expression (Table 3).

**TABLE 3:** Summary of the effects exerted by the three different drugs on alphaB-crystallin and GFAP expression levels and resulting effects on GFAP R239C aggregates.

	Endogenous alphaB-crystallin		Endogenous GFAP			Mutant GFAP elimination	Effect on mutant GFAP aggregates
	mRNA	protein	Transcription	mRNA	protein		
PHT	NO	NO	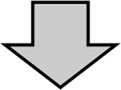	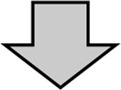	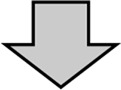	NO	ND
CBZ	ND	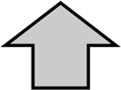	NO	ND	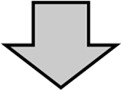	NO	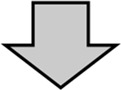
CLM	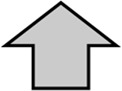	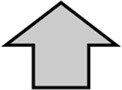	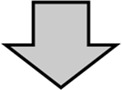	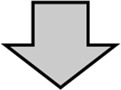	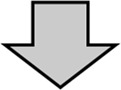	NO	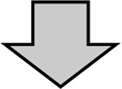

NO: no effect; ND: not detected.

Conversely, no modifications in vimentin distribution were observed following treatments as vimentin correctly assembled into aggresomes structures as expected in the presence of large perinuclear mutant GFAP inclusions and in filamentous patterns in the presence of regular GFAP conformation.

Thus, while PHT is very effective in regulating *GFAP* gene expression, CBZ is able to induce a correct refolding, likely through induction of HSPs as it occurs for CLM, which shares a similar tricyclic structure with CBZ. On the other hand, with respect to PHT, CLM seems to be the molecule acting through a wider range of mechanisms, positively affecting both GFAP expression and HSPs mediated mutant GFAP refolding.

**FIGURE 8:**
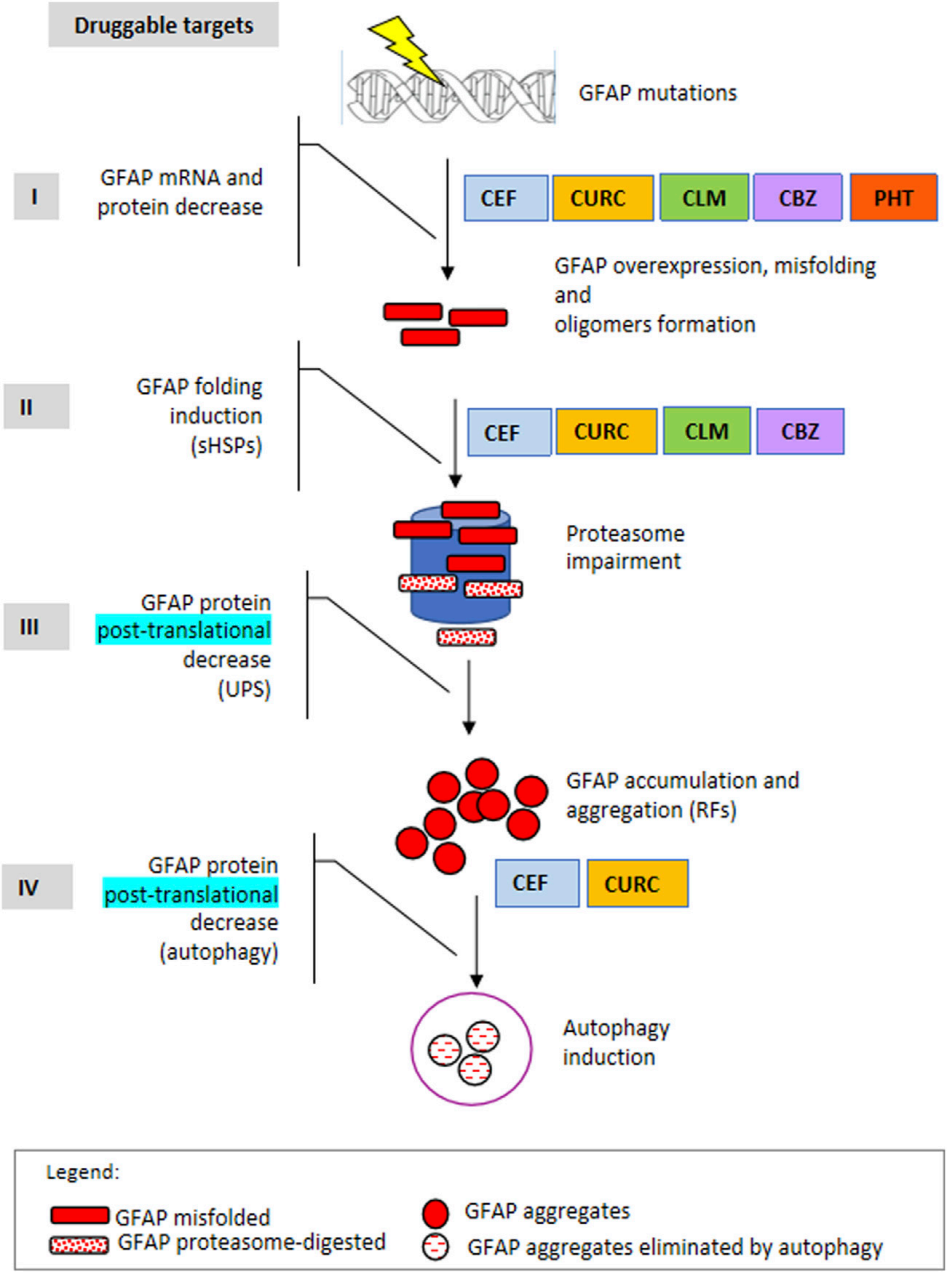
Druggable targets in Alexander disease. The scheme represents the four main steps of gene expression regulation as druggable targets to counteract mutant GFAP aggregation. I: mRNA and protein level, in terms of transcriptional and/or posttranscriptional regulation; II: protein folding; III: protein elimination mediated by the ubiquitin-proteasome system; IV: protein elimination mediated by autophagy. In addition, molecules identified so far by us to act in one or more processes are indicated on the right.  CLM: clomipramine, CBZ, carbamazepine; PHT: 5,5-diphenylhydantoin, CURC: curcumin, CEF: ceftriaxone.

Similar to other repurposed compounds previously evaluated for AxD, such as curcumin and ceftriaxone (Bachetti et al., 2010b; Bachetti et al., 2012), the molecules taken into consideration in the present work have displayed a variable range of beneficial effects, including the induction of HSP27 and alphaB-crystallin cellular expression, reduction of cellular GFAP levels by interfering with its gene expression, and elimination of the p. R239C mutant protein (Figure 8). Importantly, ceftriaxone has also been used to treat AxD in one patient with an adult, rapidly progressive form of the pathology, and this treatment apparently halted the progression of the disease and ameliorated the neurological features (Sechi et al., 2010; Sechi et al., 2013). Such an observation, along with the identification of further molecules displaying putatively beneficial effects, encourages the establishment of a deeper benefit/risk profile of similar compounds in AxD.

## Conclusion

Similar to CLM, a powerful proconvulsant drug, the antiepileptic agents PHT and CBZ have shown a definite beneficial effect in a reliable *in vitro* model of AxD. CBZ, in particular, which has a very safe profile, has been revealed to be the most effective treatment in rescuing the correct pattern of mutant GFAP in the model. These findings may have important implications for better understanding the pharmacology of AxD and other neurodegenerative disorders caused by different misfolded proteins (Sechi et al., 2011; Ruzza et al., 2014) in particular for qualitative/quantitative structure-activity relationship studies. Moreover, our observations have a considerable clinical relevance because of the safer profile of CBZ compared to CLM and PHT in clinical practice.

As CBZ and PHT are currently approved for use in humans, the documented inhibitory effect of these antiepileptic agents on pathological GFAP cellular expression and folding may indicate their potential therapeutic role as disease-modifying agents in the clinical management of AxD, particularly in AxD patients with focal epilepsy with and without secondary generalization.

## Data Availability

The original contributions presented in the study are included in the article/supplementary material; further inquiries can be directed to the corresponding authors.
